# Genomic selection across multiple breeding cycles in applied bread wheat breeding

**DOI:** 10.1007/s00122-016-2694-2

**Published:** 2016-04-11

**Authors:** Sebastian Michel, Christian Ametz, Huseyin Gungor, Doru Epure, Heinrich Grausgruber, Franziska Löschenberger, Hermann Buerstmayr

**Affiliations:** Department for Agrobiotechnology (IFA-Tulln), Institute for Biotechnology in Plant Production, University of Natural Resources and Life Sciences, Vienna (BOKU), Konrad-Lorenz-Str. 20, 3430 Tulln, Austria; Saatzucht Donau GesmbH. and CoKG, Saatzuchtstrasse 11, 2301 Probstdorf, Austria; ProGen Seed A.Ş., Büyükdalyan Mah. 2. Küme evler Sok., No: 49, Antakya/Hatay, Turkey; Probstdorfer Saatzucht Romania SRL, Str. Siriului Nr.20, sect. 1, Bucharest, Romania; Plant Breeding Division, Department of Crop Science, University of Natural Resources and Life Sciences, Vienna (BOKU), Konrad-Lorenz-Str. 24, 3430 Tulln, Austria

## Abstract

*****Key message***:**

**We evaluated genomic selection across five breeding cycles of bread wheat breeding. Bias of within-cycle cross-validation and methods for improving the prediction accuracy were assessed.**

**Abstract:**

The prospect of genomic selection has been frequently shown by cross-validation studies using the same genetic material across multiple environments, but studies investigating genomic selection across multiple breeding cycles in applied bread wheat breeding are lacking. We estimated the prediction accuracy of grain yield, protein content and protein yield of 659 inbred lines across five independent breeding cycles and assessed the bias of within-cycle cross-validation. We investigated the influence of outliers on the prediction accuracy and predicted protein yield by its components traits. A high average heritability was estimated for protein content, followed by grain yield and protein yield. The bias of the prediction accuracy using populations from individual cycles using fivefold cross-validation was accordingly substantial for protein yield (17–712 %) and less pronounced for protein content (8–86 %). Cross-validation using the cycles as folds aimed to avoid this bias and reached a maximum prediction accuracy of $r_{\text{GS}}$ = 0.51 for protein content, $r_{\text{GS}}$ = 0.38 for grain yield and $r_{\text{GS}}$ = 0.16 for protein yield. Dropping outlier cycles increased the prediction accuracy of grain yield to $r_{\text{GS}}$ = 0.41 as estimated by cross-validation, while dropping outlier environments did not have a significant effect on the prediction accuracy. Independent validation suggests, on the other hand, that careful consideration is necessary before an outlier correction is undertaken, which removes lines from the training population. Predicting protein yield by multiplying genomic estimated breeding values of grain yield and protein content raised the prediction accuracy to $r_{\text{GS}}$ = 0.19 for this derived trait.

**Electronic supplementary material:**

The online version of this article (doi:10.1007/s00122-016-2694-2) contains supplementary material, which is available to authorized users.

## Introduction

Originally suggested by Meuwissen et al. (2001), genomic selection showed great promise to strongly increase the rate of genetic improvement in both animal and plant breeding programs. This new method allowed a comparative larger gain from selection by estimating all marker effects simultaneously and subsequent selection of genetically superior individuals based on their genomic estimated breeding value (GEBV) (Bernardo and Yu 2007; Piyasatian et al. 2007), instead of using a few significant markers as in classical marker-assisted selection (Lande and Thompson 1990). Genomic selection was readily integrated into applied animal breeding programs due to a high accuracy of breeding values and a previously existing similar system, which employed pedigree instead of marker information (VanRaden 2008; Hayes et al. 2009). Notwithstanding, the relative simple methodology made genomic selection also interesting for applied plant breeding: a training population of phenotyped and genotyped individuals is utilized to establish a statistical model that predicts breeding values of non-phenotyped individuals from a selection or validation population by their genomic fingerprints (Heffner et al. 2009; Jannink et al. 2010).

Although methodologically simple the sparse knowledge about its functionality made it initially difficult to find starting points for increasing the prediction accuracy. Theoretical studies thus laid the foundation for optimizing breeding with genomic selection by trying to understand the underlying mechanics of this ‘green box’ approach. The driving forces of prediction accuracy that can be most readily influenced by plant breeders are the training population size and heritability (Muir 2007; Hayes et al. 2009), by adequately adjusting the resource allocation (Riedelsheimer and Melchinger 2013; Longin et al. 2015). Recent advances in sequencing technologies made it possible to apply cost effective genotyping methods such as genotyping-by-sequencing (GBS) in various crop species (Elshire et al. 2011; Poland et al. 2012; Huang et al. 2014) yielding an appropriate large number of markers for genomic selection (Hayes et al. 2009; Schulz-Streeck et al. 2011). The use of dense genome-wide markers increases the chance of markers being in linkage disequilibrium (LD) with QTL influencing the trait of interest (e.g. Meuwissen et al. 2001), and determines to some extent how well genetic relationship and genetic architecture are captured by the genomic selection model (Daetwyler et al. 2010; Heslot et al. 2013a). The importance of a close genetic relationship between training and validation populations to achieve a high prediction accuracy (Habier et al. 2013) has been verified numerous times in plant breeding studies, e.g. with sugar beet (Würschum et al. 2013); rapeseed (Würschum et al. 2014), maize (Zhao et al. 2012; Riedelsheimer et al. 2013; Albrecht et al. 2014; Lehermeier et al. 2014), and wheat (Charmet et al. 2014; Crossa et al. 2014), which motivated investigations for an optimal training population construction to reduce phenotyping costs (Rincent et al. 2012; Isidro et al. 2015).

Summing up, valuable insights into genomic selection have been gained in relative short time opening up numerous possibilities for its implementation into the framework of plant breeding (Heslot et al. 2015). Notwithstanding, most studies were conducted with the same genetic material across multiple environments or made use of historical phenotypic data (Dawson et al. 2013; Storlie and Charmet 2013; Ly et al. 2013; Rutkoski et al. 2015), while few have focused on the problem of predicting across breeding cycles in applied plant breeding programs. This issue was addressed for the first time in sugar beet breeding, where genomic selection showed great promise across two subsequent breeding cycles especially for highly heritable traits (Hofheinz et al. 2012). A population of parental lines was employed to predict GEBVs for five successive years in a recent study with six-row barley by Sallam et al. (2015), who reported varying degrees of prediction accuracy depending both on the progeny set and trait. We are not aware of any studies investigating genomic selection across multiple breeding cycles in applied bread wheat breeding; thus the objectives of this study were (1) to estimate the accuracy when predicting grain yield, protein content and protein yield of wheat inbred lines across multiple independent breeding cycles; (2) compare within-cycle and between-cycle prediction accuracy obtained from different cross-validation schemes; and (3) investigate model independent possibilities to increase the prediction accuracy.

## Materials and methods

### Plant material and phenotypic data

We analyzed five breeding cycles from a commercial winter wheat (*Triticum aestivum* L.) breeding program, where breeding cycles correspond to the different starting years. A total of 659 genotyped lines from multiple families, either *F*_4:6_ or directly derived by the double haploid method, were tested in multi-environment trials from 2010 to 2014. A balanced subset of trial locations was selected for evaluating the merit of genomic selection across multiple breeding cycles. Within each breeding cycles a different set of 64–176 lines was tested orthogonally across all trial locations. Grain yield (dt ha^−1^), protein content (%) and protein yield (dt ha^−1^) were assessed in 2–8 trials per breeding cycles. Protein content was determined by near infrared spectroscopy (NIRS) directly at harvest and protein yield was derived by multiplication of grain yield and protein content on a plot basis. An additional independent set of 178 genotyped lines from the same breeding population was tested in 2015 employing the same phenotyping procedure as in 2010–2014. Trial locations spanned from Austria over Serbia, Croatia, Hungary, and Romania to the Central Anatolian High Plateau in Turkey, thus covering a large diversity of target environments. Trials were designed according to the standard procedure in plant breeding, where non-replicated earlier generation lines are tested along with replicated checks, which allowed correcting for spatial field trends and estimation of error variances.

### Phenotypic analysis

We followed a two-stage analysis strategy of the phenotypic data, where each individual trial, i.e. location by breeding cycle combination was analyzed separately in the first stage. A baseline model without correction for spatial trend was compared by Akaike’s Information Criterion (AIC) with models correcting for row and/or column effects, if feasible an autoregressive variance–covariance structure of the residuals was additionally integrated (Burgueno et al. 2000). The model with the smallest AIC was chosen to derive best linear unbiased estimates (BLUE) for each trial. The heritability was computed as suggested by Piepho and Möhring (2007) by $h^{2} = \sigma_{\text{G}}^{2} /(\sigma_{\text{G}}^{2} + \frac{1}{2}{\text{MVD}})$, where $\sigma_{\text{G}}^{2}$ designates the genetic variance and MVD the mean variance of a difference of the BLUEs. The analysis at the first stage contained both checks and genotyped lines.

We retained only trials with a heritability larger than 0.3 and genotyped lines for the analysis across trials at the second stage. A linear mixed model of the form

1 $$y_{\text{ij}} = \mu + g_{\text{i}} + t_{\text{j}} + gt_{\text{ij}} + e$$

was fitted for all traits, where $y_{\text{ij}}$ are the BLUEs from the first stage, $\mu$ is the grand mean, and $g_{\text{i}}$ is the effect of the ith inbred line. The effect of the *j*th trial $t_{\text{j}}$ was fixed, while the line by trial interaction effect $gt_{\text{ij}}$ was random. The inverse of the squared standard errors of the means derived from the first stage of analysis were used as weights in this stage to take the varying accuracy of phenotypic records into account (Möhring and Piepho 2009). The residual variance was fixed to 1 for grain yield and 0.1 for protein content and protein yield, which allowed the separation of residual and line by trial interaction variances. Heritability estimates across trials were computed using the standard formula $h^{2} = \sigma_{\text{G}}^{2} /(\sigma_{\text{G}}^{2} + t^{ - 1} \sigma_{\text{GT}}^{ 2} )$, dividing the line by trial interaction variance $\sigma_{\text{GT}}^{2}$ by the number of trials t. All phenotypic analyses were conducted using the statistical package ASReml 3 (VSN International 2015) for the R programming environment (R development core team 2015).

### Genotypic data

Leaves for DNA extraction were sampled from *F*_4:5_ plants in small observation plots after phenotypic pre-selection during early summer. DNA was extracted following the protocol by Saghai-Maroof et al. (1984). All 659 lines were genotyped with approximately 20 K SNP markers using the DarT genotyping-by-sequencing (GBS) approach (Diversity Array Technologies, 2015). Quality control was applied by filtering out markers with a call rate lower than 90 %, a minor allele frequency smaller than 0.05, and more than 10 % of missing data. Missing data of the remaining 9.5K SNP markers was imputed by the MVN-EM algorithm by Poland et al. (2012) which was specially designed for the use of GBS markers.

### Genomic selection and genetic relationship

Genomic predictions of inbred lines were estimated using a ridge regression best linear unbiased prediction (RR-BLUP) model:

2 $${\mathbf{y}} = {\mathbf{Xb}} + {\mathbf{Zu}} + {\mathbf{e}}$$

where ${\mathbf{y}}$ is an *N* × 1 vector of BLUEs obtained in the phenotypic analysis, ${\mathbf{b}}$ is a vector of *F* fixed effects and ${\mathbf{X}}$ its corresponding *N* × *F* design matrix. ${\mathbf{Z}}$ is a *N* × *M* matrix, which coded the M markers as either +1 or −1 for homozygous loci and 0 for heterozygous loci. Random marker effects were assumed to follow a normal distribution ${\mathbf{u}} \sim N(0, {\mathbf{I}}\sigma_{\text{u}}^{2} )$ and equally shrunken towards zero given the penalty parameter $\lambda^{2} = \sigma_{\text{e}}^{2} /\sigma_{\text{u}}^{2}$ where $\sigma_{\text{e}}^{2}$ is the variance of the residuals which followed ${\mathbf{e}} \sim N(0, {\mathbf{I}}\sigma_{\text{e}}^{2} )$. The kinship between lines was estimated by the genomic relationship matrix, which was computed according to Endelman and Jannick (2012):

3 $${\mathbf{K}} = {\mathbf{WW}}^{\text{T}} /2\varSigma (p_{\text{k}} - 1)p_{\text{k}}$$

where ${\mathbf{W}}$ is a centered *N* × *M* marker matrix of the i lines with $W_{ik} = Z_{ik} - 2p_{k}$ and ${\text{p}}_{\text{k}}$ being the allele frequency at the *k*th locus. The derived variance–covariance matrix was used to fit a genomic best linear unbiased prediction (G-BLUP) model:

4 $${\mathbf{y}} = {\mathbf{Xb}} + {\mathbf{g}} + {\mathbf{e}}$$

where ${\mathbf{g}}$ is an *N* × 1 vector of genotypic effects with ${\mathbf{g}} \sim N(0, {\mathbf{K}}\sigma_{G}^{2} )$. Model (4) has been shown to be equivalent to (2) (VanRaden 2008; Piepho 2009) and allowed estimating the accuracy of each individual line by $r_{\text{PEV}} = \sqrt {1 - ({\text{PEV}}/{\mathbf{G}}_{\text{ii}} \sigma_{\text{G}}^{2} )}$ where ${\text{PEV}}$ is the prediction error variance, $\sigma_{\text{G}}^{2}$ the genetic variance explained by the model and ${\mathbf{G}}_{\text{ii}}$ is the diagonal element of the genomic relationship matrix for each line *i* (Clark et al. 2012). All models for genomic selection were implemented with the R package rrBLUP (Endelman 2011).

### Validation and accuracy

At first we estimated the correlation between the accuracy of each individual line $r_{\text{PEV}}$ and the genetic relationship to investigate this important driving force of prediction accuracy across several cycles of wheat breeding. The average genetic relationship of the most related lines from the training population was computed for each line in the validation population and correlated with $r_{\text{PEV}}$. The number of most related lines was varied between 1 and 500, and one breeding cycle was left out at a time using all other breeding cycles as training population. A fixed year effect was included into model (4) to account for the different yield levels of the studied breeding cycles. Prediction accuracy is generally defined as the Pearson correlation between predicted and true breeding values $r_{\text{MT}} = r_{{{\text{GEBV}}, {\text{TBV}}}}$. The true breeding values were unknown in our study; so we estimated prediction accuracy as the correlation between predicted and observed line performance $r_{\text{GS}} = r_{{{\text{GEBV}}, {\text{BLUE}}}}$. Across-cycle prediction accuracy was subsequently assessed by computing marker effect estimates with the RR-BLUP model of all possible pair-wise training and validation population combinations of the five breeding cycles. Three cross-validation schemes each with fivefolds and 100 replicates were employed to cover different aspects of the prediction accuracy $r_{\text{GS}}$:

1. Within-cycle prediction accuracy was computed by randomly dividing the data into equally sized folds using 80 % of lines within each breeding cycle as a training population and subsequent prediction of the left-out fold. This procedure was repeated for every fold and the resulting prediction accuracy was averaged for each of the 100 replicates.
2. The same training populations as in the within-cycle cross-validation were used to separately predict lines of each other breeding cycle. The average prediction accuracy was saved and utilized to estimate the bias of within-cycle versus between-cycle cross-validation.
3. Fivefold cross-validation, where the breeding cycles constituted the folds, was used to estimate the prediction accuracy across cycles. An equal number of lines were randomly sampled from each breeding cycle, simulating a breeding scenario where training populations for genomic selection models are an assembly of several mixed populations from multiple breeding cycles. Training population sizes varied between 16 and 256 lines. An additional fixed year effect was added to model (2) in order to account for the different yield and protein levels in 2010 to 2014.

Furthermore we studied two possibilities for increasing the prediction accuracy across breeding cycles. First outliers were identified by approximating the genetic correlation among environments by their pair-wise prediction accuracies (Heslot et al. 2013b), and breeding cycles or trials with a strongly deviating character were dropped from the training population. The influence of these outliers was subsequently investigated by comparing the prediction accuracy with the full and outlier corrected dataset, using the same across-cycle cross-validation approach as before. Training population size was kept constant by randomly choosing additional lines from each remaining breeding cycle in the outlier corrected cross-validation. In addition to cross-validation, the 178 lines from 2015 served as an independent validation population and were predicted by estimated marker effects using either the full or outlier corrected dataset.

Finally we investigated the possibility to increase the prediction accuracy of the derived trait protein yield by multiplying GEBVs of its component traits grain yield and protein yield. The prediction accuracy was estimated by the above described across-cycle cross-validation approach with 100 replicates for each training population size.

## Results

### Quantitative-genetic parameters

The plant material was tested in a broad spectrum of environments ranging from the Pannonian Basin to the Central Anatolian High Plateau. Despite the expected large genotype by environments interaction we observed a medium to high heritability in each individual breeding cycle for grain yield and protein content (Table 1). A relatively large number of trials having at least a heritability larger than 0.3 were pre-selected for this study to achieve valid and robust results. The excellent data quality was also reflected by the medium to high heritability for protein yield in all but one breeding cycle. Estimates of heritability were lower for protein yield than grain yield except for 2013, where it was 26 % larger. The protein content had on average the highest heritability followed by grain yield and protein yield.

**Table 1:** Mean, variance components and heritability for grain yield (dt ha^−1^), protein content (%) and protein yield (dt ha^−1^) of genotyped lines across all trials in the respective breeding cycles 2010–2014

Trait	Parameter	Breeding cycles				
		2010	2011	2012	2013	2014
Grain yield	Trials	5	6	4	5	8
	$\sigma_{\text{G}}^{2}$	2.28 ± 1.28	4.60 ± 1.60	5.03 ± 1.25	6.64 ± 1.76	37.00 ± 4.71
	$\sigma_{\text{GT}}^{2}$	23.70 ± 1.83	23.67 ± 1.99	17.80 ± 1.21	40.98 ± 2.36	54.48 ± 2.26
	*h* ^2^	0.32	0.54	0.53	0.45	0.84
Protein content	Trials	4	2	3	4	2
	$\sigma_{\text{G}}^{2}$	0.23 ± 0.05	0.18 ± 0.05	0.35 ± 0.05	0.37 ± 0.06	0.33 ± 0.09
	$\sigma_{\text{GT}}^{2}$	0.36 ± 0.04	0.07 ± 0.03	0.27 ± 0.03	0.65 ± 0.05	0.65 ± 0.08
	*h* ^2^	0.72	0.84	0.80	0.69	0.50
Protein yield	Trials	4	2	4	4	3
	$\sigma_{\text{G}}^{2}$	0.04 ± 0.03	0.03 ± 0.07	0.05 ± 0.02	0.26 ± 0.05	0.76 ± 0.14
	$\sigma_{\text{GT}}^{2}$	0.41 ± 0.04	0.38 ± 0.09	0.34 ± 0.03	0.69 ± 0.05	1.30 ± 0.11
	*h* ^2^	0.30	0.14	0.37	0.60	0.64
	Lines	94	64	165	160	176

Genotypic variance ($\sigma_{\text{G}}^{2}$), genotype by trial interaction variance ($\sigma_{GT}^{2}$), and heritability (*h* ^*2*^)

### Genetic relationship and prediction accuracy of genomic selection

The correlation between accuracy of each individual line and the genetic relationship was strongly dependent on the number of most related lines and the respective validation population (Fig. S1). Optimal correlations for grain yield were achieved using the 11–133 most related lines, while choosing the 70 most related lines led to significant correlations larger than *r* = 0.80 for all validation populations. Similar patterns were observed for protein content and protein yield. The average of the top 70 genetic relationship between lines range from 0.08 to 0.14 within the years, and was smaller between years with an overall average genetic correlation of 0.07 (Fig. S2).

**Fig. 1:**
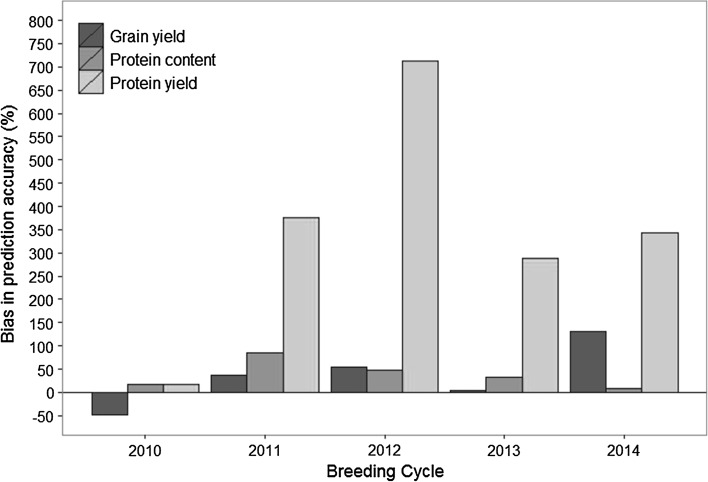
Bias of the within- cycle prediction accuracy in comparison with the between-cycle prediction accuracy for grain yield, protein content and protein yield and using lines from the years 2010–2014 as training populations

Within-cycle prediction accuracy was compared to between-cycle prediction accuracy by fivefold cross-validation utilizing the same training populations for each cross-validation scheme. A strong upward bias of within-cycle prediction accuracy was observed for 10 out of 15 traits by cycle combinations and was less than 25 % in four instances (Fig. 1). The bias was especially pronounced for 2014, where the predictive ability of grain yield was overestimated by 130 % and even more for protein yield by 344 %. Protein yield had overall the largest bias ranging from 17 % up to 712 %, while the prediction accuracy of protein content was maximally overestimated by 86 %. Within-cycle cross-validation underestimated the prediction accuracy for grain yield by 47 % merely in one case.

The intention behind using the breeding cycles as folds in a fivefold cross-validation was the avoidance of this bias when estimating the prediction accuracy. Sampling an equal number of lines from each breeding cycle furthermore aimed to avoid a confounding effect between training population sizes and breeding cycle. According to expectation the prediction accuracy increased with the number of lines in the training population (Fig. S3). A maximum was reached for a training population size of 240 lines at $r_{\text{GS}}$ = 0.51 for protein content, $r_{\text{GS}}$ = 0.38 for grain yield and $r_{\text{GS}}$ = 0.16 for protein yield.

### Outlier correction and estimation of derived traits

**Fig. 2:**
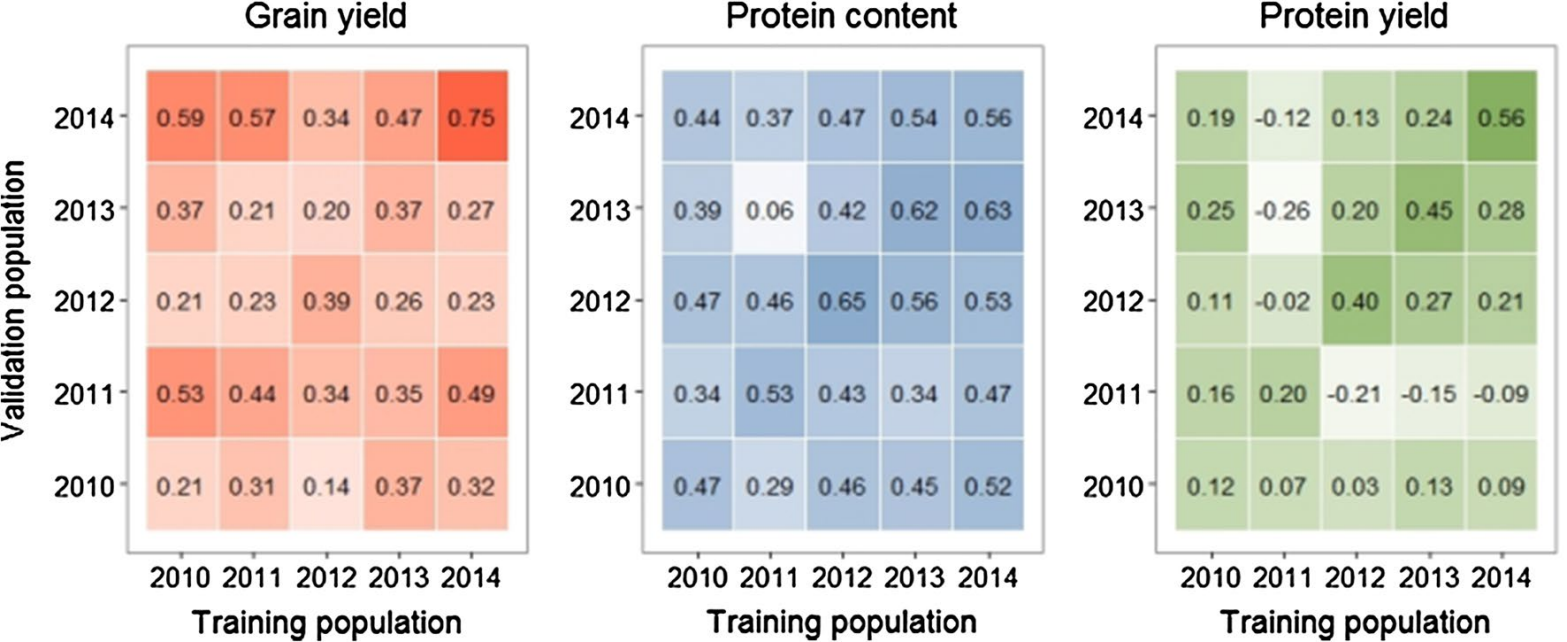
Heatmap of the pair-wise prediction accuracy between breeding cycles on the off-diagonal and the result of the fivefold within-cycle cross-validation on the diagonal

Pair-wise prediction accuracies furthermore provided an approximation of the genetic correlation between breeding cycles. It was assumed that a breeding cycle with an overall low predictive ability also had a low value of representativeness for the breeding program across several breeding cycles, and was thus considered an outlier. The breeding cycle 2012 clearly presents itself as such an outlier with regard to grain yield. It achieved on average a much lower prediction accuracy when utilized as a training population ($r_{\text{GS}}$ = 0.26) in comparison with all other breeding cycles ($r_{\text{GS}}$ = 0.36) (Fig. 2). Although the heritability was relatively high for 2012, the phenotypic data was most likely strongly influenced by frost damage and drought stress in some trials. The low predictability and prediction accuracy furthermore identified the breeding cycle 2011 as an outlier for protein yield.

**Fig. 3:**
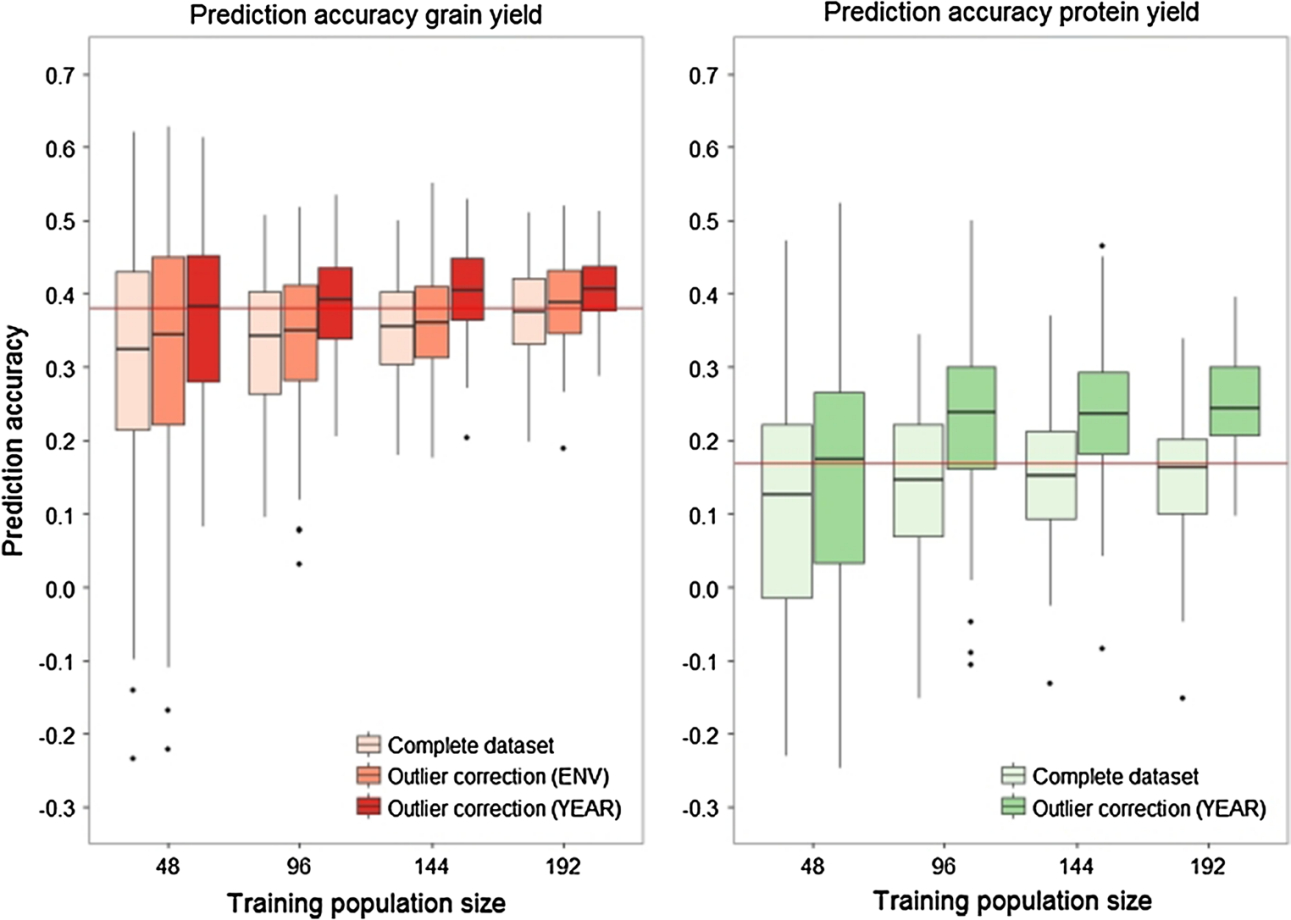
Influence of removing outlier years or environments from the training set on the prediction accuracy. Results were obtained using across-cycle cross-validation with years as folds. The *horizontal red line* indicates the maximum prediction accuracy in the complete dataset

The influence of these outliers was investigated by omitting the above described breeding cycles when selecting lines for the training population. Using this approach, the prediction accuracy of protein yield increased from $r_{\text{GS}}$ = 0.15 to $r_{\text{GS}}$ = 0.25 at a training population size of 192 lines (Fig. 3). A similar pattern was observed for grain yield at the same training population size, where the prediction accuracy raised from $r_{\text{GS}}$ = 0.38 to $r_{\text{GS}}$ = 0.41 when omitting phenotypic data from 2012. Strikingly a prediction accuracy of $r_{\text{GS}}$ = 0.41 was estimated in the outlier corrected dataset using a training population size of 144 lines, surpassing the maximum of $r_{\text{GS}}$ = 0.38 with a much larger training population of 256 lines in the complete dataset. One of the trials suffered severe drought stress and showed a negative phenotypic correlation with all other trials from 2012, and its mean prediction accuracy as a training population for all other trials was negative ($r_{\text{GS}}$ = −0.15) and far below the average of all trials ($r_{\text{GS}}$ = 0.16). Removing this trial from the phenotypic analysis of grain yield increased the heritability to *h*^2^ = 0.61 and the average prediction accuracy of 2012 from $r_{\text{GS}}$ = 0.26 to $r_{\text{GS}}$ = 0.30, although the prediction accuracy obtained by cross-validation was not significantly higher than without outlier correction.

**Fig. 4:**
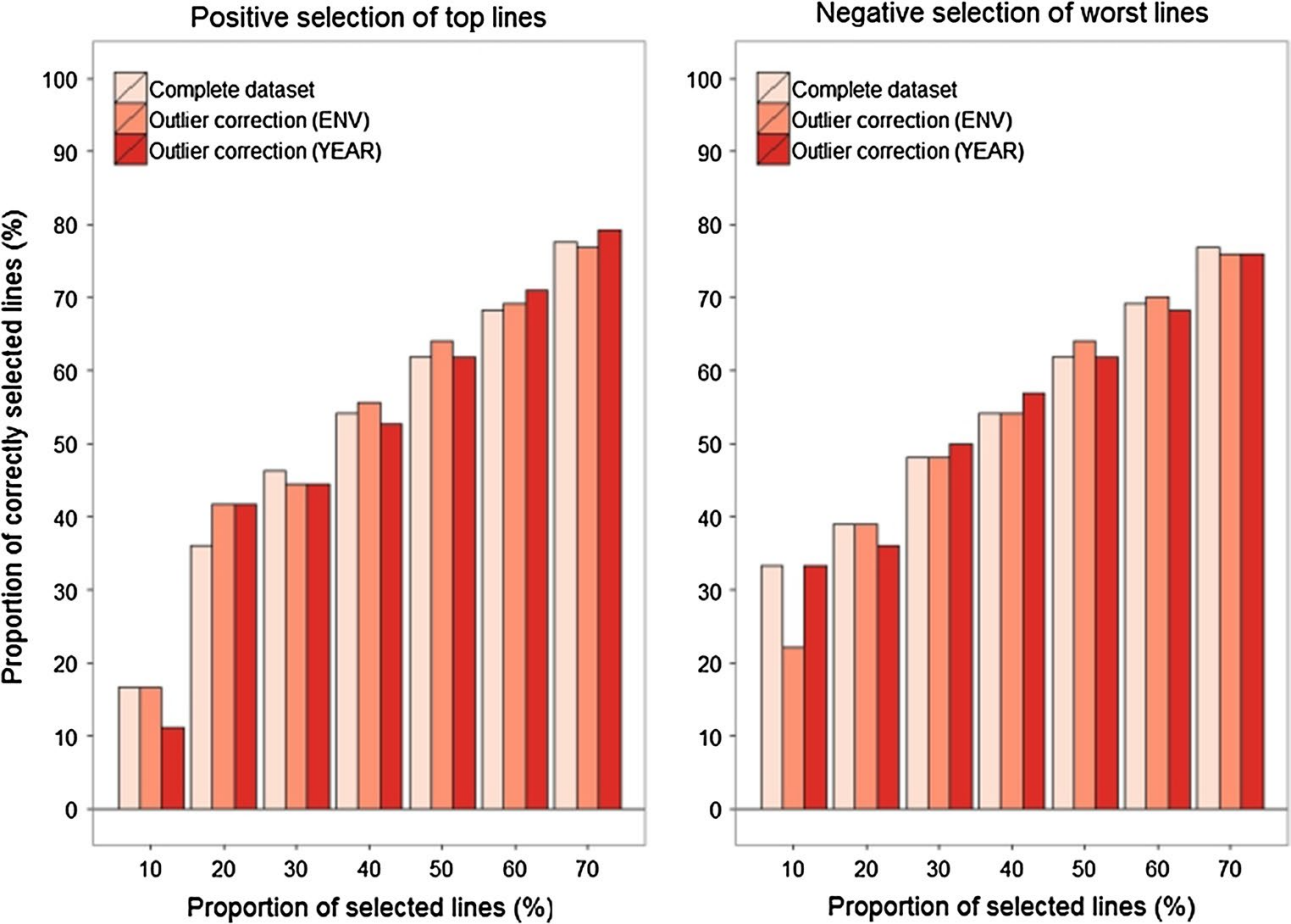
Proportion of correctly selected lines when applying genomic selection for grain yield of either the best or worst lines in the independent validation population of the year 2015

Grain yield was predicted with an accuracy of $r_{\text{GS}}$ = 0.34 in the independent validation population of the breeding cycle 2015 (*h*^2^ = 0.57). Dropping the drought stressed trial from the phenotypic analysis had no effect, while removing the entire breeding cycle 2012 decreased the prediction accuracy by 4 %. Removing 2011 from the training population decreased the prediction accuracy of protein yield by 3 % in the independent validation (*h*^2^ = 0.30). These minor changes in prediction accuracy had only a slight influence when genomically selecting for the top or against the worst lines tested in multi-environment trials in 2015 (Fig. 4).

The prediction accuracy of protein yield was rather low, while its component traits grain yield and protein content were estimated more accurately. The low estimated prediction accuracy for protein yield was raised from $r_{\text{GS}}$ = 0.16 to $r_{\text{GS}}$ = 0.19 by multiplying GEBVs of its component traits, instead of modeling protein yield directly, which corresponds to an increase of 19 %.

## Discussion

Genomic selection has received attention in plant breeding research and caused some excitement in the last years (e.g. Heffner et al. 2009; Heslot et al. 2015). Nevertheless, results from practical applications in the framework of applied breeding programs are still sparse. This study focused on the problems and prospects of genomic selection in bread wheat. Five breeding cycles from an ongoing commercial breeding program were used as a base for assessing and enhancing the potential of genomic selection in bread wheat breeding.

### Model selection

Since the introduction of genomic selection models from both the Bayesian (e.g. Technow and Melchinger 2013) and Frequentist methodology (Piepho 2009; Schulz-Streeck and Piepho 2010; Hofheinz and Frisch 2014) as well as machine learning methods (Ogutu et al. 2011, 2012) have been applied in plant breeding. Although great effort was put into developing these models no method showed clear superiority over the others across species or traits (Heslot et al. 2012). Hence we chose RR-BLUP as a computationally fast and robust alternative in our study.

### Bias of the prediction accuracy

Genomic selection of non-phenotyped genotypes based on RR-BLUP is strongly dependent on the relationship between training population and selection candidates (Habier et al. 2007; Hayes et al. 2009). Empirical studies from plant breeding show a strong decline in accuracy when predicting distantly related populations (Riedelsheimer et al. 2013; Albrecht et al. 2014) and higher accuracies within closely related families (Lehermeier et al. 2014). Hence, the genetic relationship between training and selection population might introduce a bias in the estimation of prediction accuracy depending on the cross-validation scheme (Ly et al. 2013; Würschum et al. 2013). Genotype by environment interaction effects furthermore led to overestimations of the prediction accuracy, when genotypes from the training and selection population were tested in the same environment or year (Storlie and Charmet 2013; Krchov et al. 2015).

Both effects play important roles when predicting selection candidates across cycles in an ongoing breeding program. Accordingly, Hofheinz et al. (2012) reported an upward bias of the within-cycle prediction accuracy computed by cross-validation in comparison with the actual prediction accuracy across two subsequent breeding cycles. This observation was verified by our analysis and especially pronounced for protein yield, while the bias was much smaller for protein content. The highly heritable traits fusarium head blight resistance and plant height showed likewise less variation in the prediction accuracy across several breeding cycles, than the complex and low heritable trait grain yield in a dynamic barley breeding population (Sallam et al. 2015). A similar relationship between heritability and across-cycle prediction accuracy was also found in sugar beet (Hofheinz et al. 2012). Highly heritable traits are expected to have a less complex genetic architecture (Combs and Bernardo 2013), thus haplotype structures and relatedness responsible for the prediction accuracy (Daetwyler et al. 2010) might be preserved across breeding cycles. These considerations are in accordance with the presented empirical results and suggest that prediction accuracy estimates for highly heritable traits are quite stable even across multiple breeding cycles.

### Cross-validation results

Genomic selection is though especially interesting for low heritable traits and providing realistic estimates of trait-specific parameters is crucial for optimizing the resource allocations in an applied breeding program (Riedelsheimer and Melchinger 2013; Longin et al. 2015). Implementation of genomic selection in a breeding program faces the challenge of predicting a new set of genotypes with varying relatedness to previous generations or breeding cycles every year. Using breeding cycles as folds in cross-validation takes this problem into account, by sampling non-overlapping sets of genotypes from multiple breeding cycles as training populations and subsequent prediction of an independent breeding cycle. Estimates for grain yield derived from this across-cycle cross-validation scheme were on the upper bound of what has been reported before in mixed wheat populations (Heffner et al. 2011; Poland et al. 2012; Combs and Bernardo 2013; Storlie and Charmet 2013; Charmet et al. 2014; Isidro et al. 2015). Considering these studies together with our results a prediction accuracy between 0.3 (Longin et al. 2015) and 0.4 seems to be realistic for wheat grain yield across several breeding cycles. Interestingly these values correspond to the broad-sense heritability using variance components from Piepho et al. (2014) who analyzed long-term trends of bread wheat in the German official variety trials. A similar relationship between heritability and prediction accuracy across breeding cycles was previously observed by Hofheinz et al. (2012) for sugar content and molasses loss in sugar beet and several traits in barley (Sallam et al. 2015).

Particular with regard to the importance of phenotypic data (Bernal-Vasquez et al. 2014) a high estimate of the prediction accuracy was not unexpected in this study. First we selected trials with a high heritability from a larger population of target environments, as is common practice in plant breeding. Additionally, we selected only phenotypic records of lines that were tested orthogonally across all trials within a breeding cycle for building training populations. This allowed direct comparisons between all lines and consequently an expected higher efficiency than partial comparisons between lines or by using check varieties (Piepho et al. 2006). Even if a balanced subset cannot be extracted from existing data we recommend the use of the very best of trials to compute GEBVs for pending selection decisions as the data quality, measured by the heritability, is an important driving force of prediction accuracy in genomic selection (Jannink et al. 2010; Combs and Bernardo 2013).

### Outlier correction and estimation of derived traits

A high heritability suggests that the phenotypic accuracy is high and gives good estimates of the underlying genotypes and true breeding values of the selection candidates (Visscher et al. 2008). Nevertheless, some years or trials have a low predictability despite of high heritability estimates (Dawson et al. 2013). Factors like biotic or abiotic stress caused by heavy rain, frost damage or drought can result in poor trial establishment and characterizes such environments as outliers (Heslot et al. 2013b). Although they give breeders the opportunity to select for special traits e.g. winter hardiness or resistance to a specific disease, dropping such outlier environments is justified when breeding for productivity and broad adaptation.

This outlier correction increased the prediction accuracy of grain yield, estimated by cross-validation, by 16 % in our study. We used pair-wise prediction accuracies and breeder´s knowledge as an ad hoc measure to identify outliers for grain yield in wheat, though implementation of a systematic search algorithm led to analogous results for grain yield in barley (Heslot et al. 2013b). Dropping low-quality data for protein yield had a similar effect by raising the prediction accuracy by 50 % underpinning again the importance of phenotypic data. Independent validation suggests on the other hand that careful consideration is necessary before an outlier correction is undertaken. Dropping all phenotypic records of a genotype might even have a detrimental effect on the prediction accuracy in some cases as a broad genetic base and maximizing the phenotypic variance are essentials for optimizing a training population (Rincent et al. 2012; Isidro et al. 2015).

Apart from outlier correction another convenient option to improve the prediction accuracy for the derived trait protein yield was its prediction by component traits. The low prediction accuracy of protein yield could be slightly raised by multiplying GEBVs of the medium predictable traits grain yield and protein content. This approach might also be beneficial for other derived traits in plant breeding with a low heritability or prediction accuracy.

## Conclusions

Numerous genomic selection studies were conducted in recent years, pointing out its large potential and several applied plant breeding programs adopted this new technology with high expectations. Hence results from multiple genomically selected breeding cycles are becoming available now, bringing these expectations to a realistic level. Genomic selection certainly opened up new opportunities by predicting difficult or expensive to phenotype traits or the estimation of derived traits by GEBVs of its components. Furthermore the genomic selection framework helped to shed light on old problems, such as handling phenotypic data by approximating the genetic correlations among environments by their pair-wise prediction accuracy. Finally it also demands solutions to new problems such as optimizing training populations or redesigning breeding programs. Supported by the vast ongoing research, genomic selection is definitively becoming an integral part of modern bread wheat breeding and the future genetic improvement of crop plants.

### Author contribution statement

SM and CA analyzed the data and wrote the manuscript. HGR supported in the statistical analysis. FL, DE and HGU designed the field trials and collected the phenotypic data. FL and HB initiated and guided through the study. All authors read and approved the final manuscript.

## Electronic supplementary material

Below is the link to the electronic supplementary material.

Supplementary material 1 (PDF 100 kb)

Supplementary material 2 (PDF 217 kb)

Supplementary material 3 (PDF 88 kb)
