# Supplementary material for: Genomic selection across multiple breeding cycles in applied bread wheat breeding
Source: Theor Appl Genet. 2016 Apr 11;129:1179–89. doi: 10.1007/s00122-016-2694-2 (PMC4869760; doi:10.1007/s00122-016-2694-2)
Supplement: Supplementary file 1 — Supplementary material 1 (PDF 100 kb) [file 122_2016_2694_MOESM1_ESM.pdf]

Online Resource 1

Article Title: Genomic Selection across Multiple Breeding Cycles in Applied Bread Wheat Breeding

Journal: Theoretical and Applied Genetics

Authors: Sebastian Michel, Christian Ametz, Huseyin Gungor, Doru Epure, Heinrich Grausgruber, Franziska Löschenberger, Hermann Buerstmayr

Name, affiliation, and email of corresponding author:

Hermann Buerstmayr
Department for Agrobiotechnology (IFA-Tulln)
Institute for Biotechnology in Plant Production
University of Natural Resources and Life Sciences, Vienna (BOKU)
Konrad-Lorenz-Str. 20, 3430 Tulln, Austria
e-mail: hermann.buerstmayr@boku.ac.at

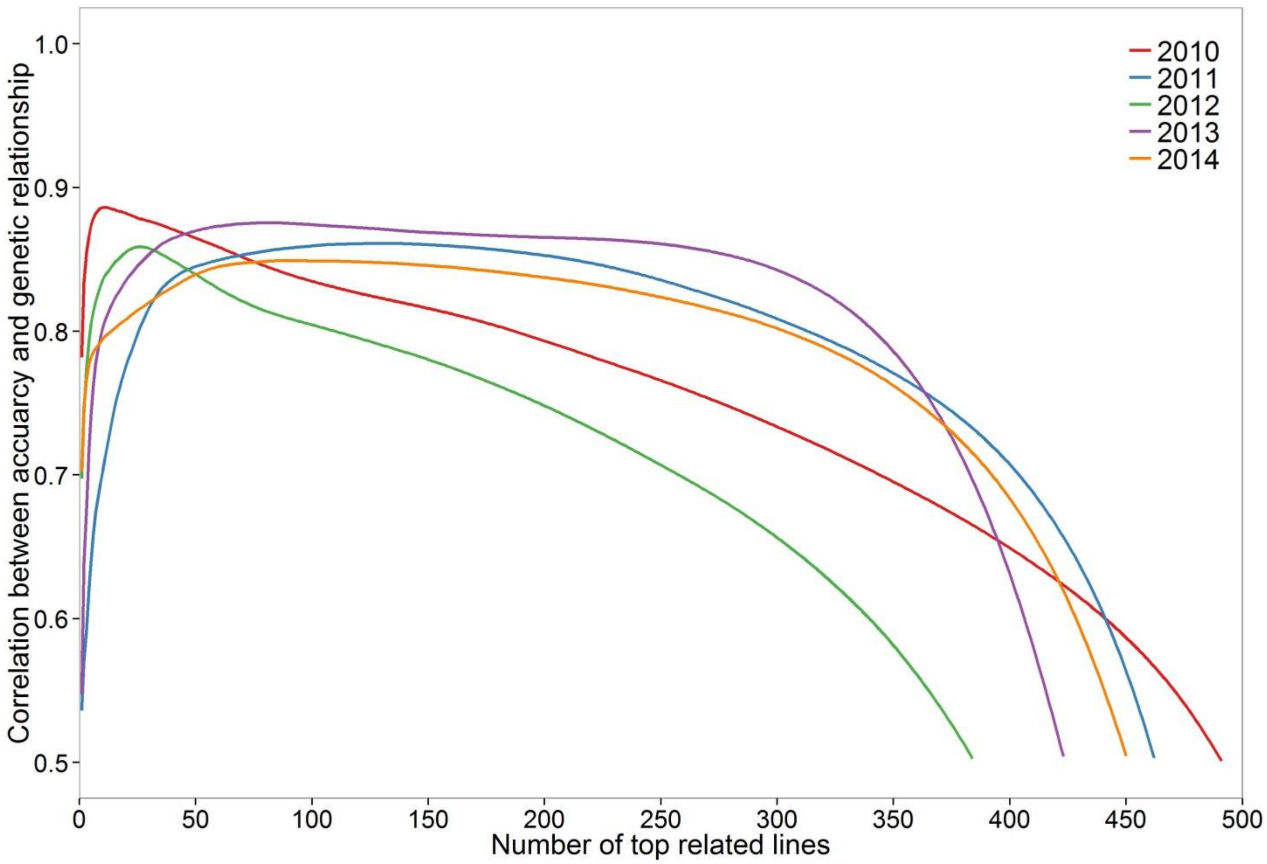

Fig. S1 Correlation between the accuracy of each individual line and the genetic relationship, varying the number of the most related lines from the training population. One breeding cycle was left out at a time using all other breeding cycles as training population.
