# Supplementary material for: Genomic selection across multiple breeding cycles in applied bread wheat breeding
Source: Theor Appl Genet. 2016 Apr 11;129:1179–89. doi: 10.1007/s00122-016-2694-2 (PMC4869760; doi:10.1007/s00122-016-2694-2)
Supplement: Supplementary file 3 — Supplementary material 3 (PDF 88 kb) [file 122_2016_2694_MOESM3_ESM.pdf]

Online Resource 3

Article Title: Genomic Selection across Multiple Breeding Cycles in Applied Bread Wheat Breeding

Journal: Theoretical and Applied Genetics

Authors: Sebastian Michel, Christian Ametz, Huseyin Gungor, Doru Epure, Heinrich Grausgruber, Franziska Löschenberger, Hermann Buerstmayr

Name, affiliation, and email of corresponding author:

Hermann Buerstmayr
Department for Agrobiotechnology (IFA-Tulln)
Institute for Biotechnology in Plant Production
University of Natural Resources and Life Sciences, Vienna (BOKU)
Konrad-Lorenz-Str. 20, 3430 Tulln, Austria
e-mail: hermann.buerstmayr@boku.ac.at

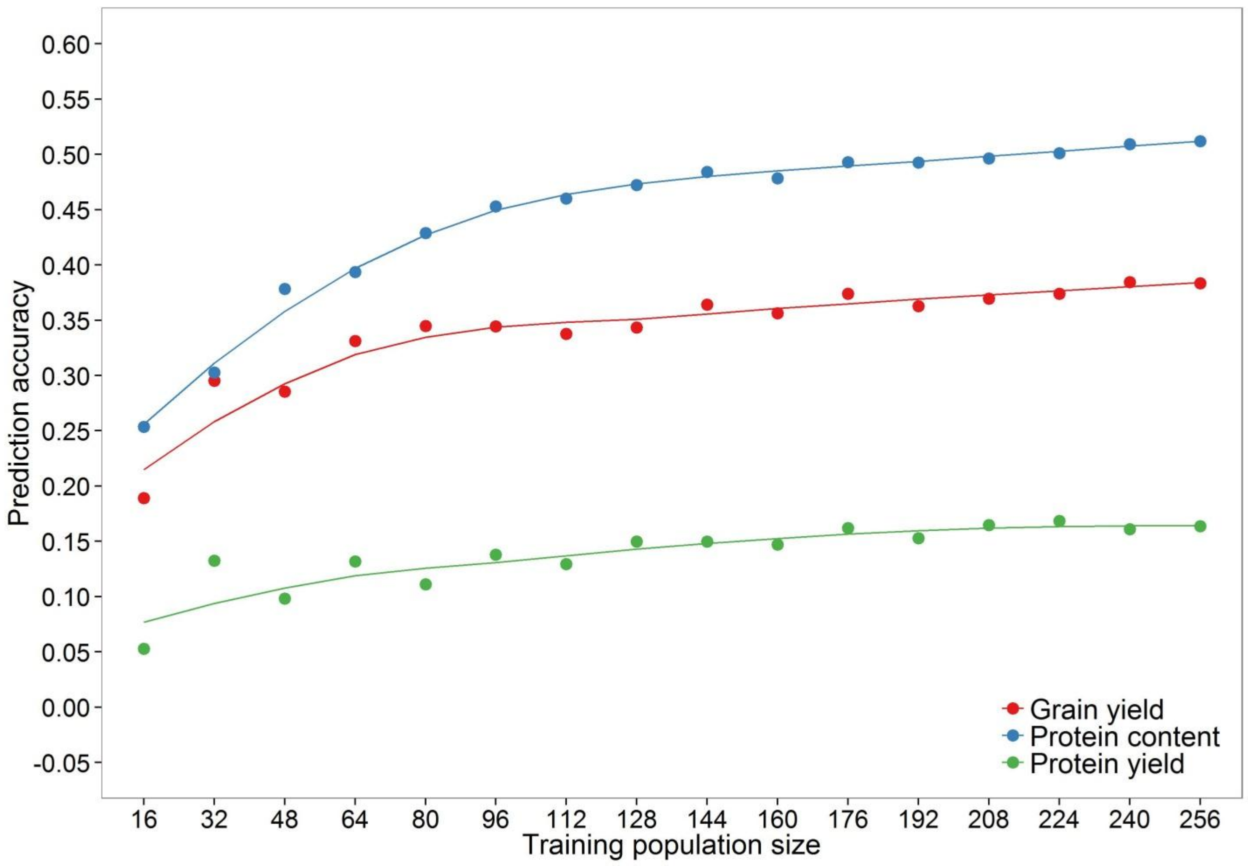

Fig. S3 Relationship between prediction accuracy and training population size for grain yield, protein content and protein yield using a 5-fold cross-validation with the breeding cycles 2010-2014 as folds.
